# Genome-Wide Association Study of Meat Quality Traits in a White Duroc×Erhualian F2 Intercross and Chinese Sutai Pigs

**DOI:** 10.1371/journal.pone.0064047

**Published:** 2013-05-28

**Authors:** Junwu Ma, Jie Yang, Lisheng Zhou, Zhiyan Zhang, Huanban Ma, Xianhua Xie, Feng Zhang, Xinwei Xiong, Leilei Cui, Hui Yang, Xianxian Liu, Yanyu Duan, Shijun Xiao, Huashui Ai, Jun Ren, Lusheng Huang

**Affiliations:** Key Laboratory for Animal Biotechnology of Jiangxi Province and the Ministry of Agriculture of China, Jiangxi Agricultural University, Nanchang, China; University of California, Davis, United States of America

## Abstract

Thousands of QTLs for meat quality traits have been identified by linkage mapping studies, but most of them lack precise position or replication between populations, which hinder their application in pig breeding programs. To localize QTLs for meat quality traits to precise genomic regions, we performed a genome-wide association (GWA) study using the Illumina PorcineSNP60K Beadchip in two swine populations: 434 Sutai pigs and 933 F2 pigs from a White Duroc**×**Erhualian intercross. Meat quality traits, including pH, color, drip loss, moisture content, protein content and intramuscular fat content (IMF), marbling and firmness scores in the M. longissimus (LM) and M. semimembranosus (SM) muscles, were recorded on the two populations. In total, 127 chromosome-wide significant SNPs for these traits were identified. Among them, 11 SNPs reached genome-wise significance level, including 1 on SSC3 for pH, 1 on SSC3 and 3 on SSC15 for drip loss, 3 (unmapped) for color a*, and 2 for IMF each on SSC9 and SSCX. Except for 11 unmapped SNPs, 116 significant SNPs fell into 28 genomic regions of approximately 10 Mb or less. Most of these regions corresponded to previously reported QTL regions and spanned smaller intervals than before. The loci on SSC3 and SSC7 appeared to have pleiotropic effects on several related traits. Besides them, a few QTL signals were replicated between the two populations. Further, we identified thirteen new candidate genes for IMF, marbling and firmness, on the basis of their positions, functional annotations and reported expression patterns. The findings will contribute to further identification of the causal mutation underlying these QTLs and future marker-assisted selection in pigs.

## Introduction

Meat quality is one of the most important economical traits in farm animals. It is decisive for the suitability of the meat for further processing and storage including retail display. The main attributes of interest are pH, color, firmness, water-holding capacity, fat content and composition, oxidative stability and uniformity [1].

Meat quality homogeneity is a major concern in the pig industry and market, but it is difficult to achieve by traditional selection because most meat quality traits exhibited low to moderate heritabilities [2], [3] and measuring them is difficult, expensive, and only possible after slaughter. Fortunately, molecular technologies have played an important role in improving meat quality. Several major genes (such as *RYR1, PRKAG3*, *IGF2*) influencing meat quality have been applied in the pig industry, resulting in considerable improvement of meat quality in commercial pig herds [4], [5].

In the past decades, quantitative trait loci (QTLs) in livestock have been detected mainly by using linkage mapping method with low-density microsatellite markers across the genome. Thus most QTLs generally span a large chromosomal region (comprising hundreds of genes), from which it is difficult to identify causative genes [6]. To date, 5,024 QTLs for meat quality traits have been deposited in pigQTLdb (http://www.animalgenome.org/cgi-bin/QTLdb/SS/), but only a handful of causative variants have been identified via QTL fine mapping analysis. During the past few years, the emergence of more cost-effective and high-throughput genotyping platforms, SNP arrays, have rendered association mapping an increasingly popular and powerful approach for QTL mapping in human, animal and plant [7]. In pigs, there is an increasing number of association studies on commercial purebreds or F2 intercross populations to detect SNPs associated with monogenetic [8] and polygenetic traits, such as hematological traits [9], [10], T lymphocyte subpopulations [11], body composition and structural soundness traits [12], boar taints [13], [14], farrowing traits [15] and meat quality traits [16], [17].

White Duroc is a lean-type western pig line and Erhualian is a Chinese fat-type indigenous pig line. They show obvious differences in meat productivity and quality, and are therefore genetically distant from each other. We have previously conducted genetic linkage analyses to detect QTLs for meat quality traits using a White Duroc**×**Erhualian F2 resource population [18], [19]. Here, we carried out GWA analyses in both the F2 population and another population: Sutai pigs. The Sutai pig is a newly developed line which contains 50% Duroc and 50% Chinese Taihu breed (including Erhualian, Meishan and Fengjing strains) and have experienced selective breeding over 18 generations. Because the founder strains of Sutai pigs are close to those of the F2 population, the objectives of this study were not only to identify the precise locations of QTLs for meat quality traits in the two populations, but also to check the consistency of QTL findings across the populations.

## Materials and Methods

### Ethics Statement

All procedures involving animals followed the guidelines for the care and use of experimental animals approved by the State Council of the People’s Republic of China. The ethics committee of Jiangxi Agricultural University specifically approved this study.

### Study Populations and Traits

A three-generation resource population and a Sutai pig population were involved in this study. The former one was created and managed from 2001 to 2006 as described by Ren et al. (2006) [20]. Briefly, two White Duroc sires and 17 Erhualian dams were mated to produce F1 animals, from which nine F1 boars and 59 F1 sows were intercrossed (avoiding full-sib mating) to produce 967 F2 males and 945 F2 females (total n = 1912) in six batches. The Sutai population comprised offspring of four boars and 55 sows. All Sutai piglets were born and raised for 2–3 months at Sutai Pig Breeding Center in Suzhou city, and then they were transferred to a farm in Nanchang city (nearby the farm used for raising the F2) at three different times (July 2, Sep. 3 and Dec. 26, 2011). Then they were fed with similar diet (formulated according to age) as that for the F2 animals under a standardized feeding and management regimen, and given free access to water. The F2 and Sutai piglets were weaned at 46 days and 28 days after birth, respectively. The castration was carried out for the F2 boars aged at 90 days and all Sutai piglets aged at 18 days including males and females.

At 240±6 days of age, a total of 1030 F2 animals including 549 gilts and 481 barrows and a total of 436 Sutai pigs including 206 gilts and 230 barrows were slaughtered at a commercial abattoir. Meat quality measurements were performed on longissimus muscle (LM) between the 10th-rib and the first lumbar vertebra and semimembranosus muscle (SM) from left-side carcass, as described in detail at elsewhere [18], [19], [21], [22]. The pH values were measured in the LM and SM by a Delta 320 pH Meter (Mettler Toledo, Greifensee, Switzerland) at 45 min and 24 h postmortem. Then, pH drop between the two time points was calculated. Meat color was subjectively assessed according to the color standard (1 = pale; 6 = dark) provided by the US National Pork Producers Council (NPPC) [23], and objectively evaluated using a CM-2600d/2500d Minolta Chroma Meter with parameters L* for lightness, a* for redness and b* for yellowness on the cut surface of the two muscles at 24 h postmortem. Drip loss after 24 h and 48 h storage of the LM and SM were measured using a bag method [24] and an EZ-DripLoss method [25]. Moisture, protein and intramuscular fat (IMF) contents of LM were determined by the routine oven-drying method, a Kjeldahl nitrogen method and an ether extraction method respectively [26]. Subjective marbling score of both muscles and firmness score of the LM were evaluated using NPPC standards [23], [27]. For the LM of Sutai, the drip loss was not measured using the bag method and the crude protein content was also not determined. In the study, 933 F2 and 434 Sutai piglets were phenotyped. Descriptive statistics of the phenotype data related to 25 traits are given in Table 1.

**Table 1:** Descriptive statistics of meat quality traits of longissimus muscle (LM) and semimembranosus muscle (SM) from a White Duroc**×**Erhualian F2 population and a Sutai (ST) population.

	F2					ST				
Traits	N	Mean	S.D.^1^	Min.	Max.	N	Mean	S.D.	Min.	Max.
**pH** 2										
LM_pH 45 min	667	6.42	0.33	5.34	7.34	378	6.51	0.49	5.41	7.82
LM_pH 24 h	673	5.67	0.17	5.35	6.71	334	5.60	0.18	5.24	6.59
LM_pHdrop_45 min_24 h	657	0.77	0.31	−0.10	1.54	298	0.78	0.38	−0.11	1.59
SM_pH 45 min	671	6.54	0.29	5.66	7.23	378	6.61	0.50	5.55	8.01
SM_pH 24 h	675	5.75	0.20	5.35	6.79	343	5.66	0.21	5.05	6.57
SM_pHdrop_45 min_24 h	669	0.78	0.31	−0.17	1.56	307	0.82	0.43	−0.11	1.71
**Meat color measures** 3										
LM_ColorM_a24 h	787	0.69	1.11	−2.57	5.67	421	0.81	1.48	−2.99	9.81
LM_ColorM_b24 h	787	7.39	1.87	2.15	13.22	421	6.21	1.78	0.01	12.23
LM_ColorM_L24 h	787	46.79	3.43	36.75	78.18	421	48.15	3.68	33.91	58.43
LM_ColorScore_24 h (1–6)	794	2.75	0.72	1.00	5.00	421	2.56	0.60	1.50	4.50
SM_ColorM_a24 h	787	3.19	1.31	−0.61	8.39	421	3.51	1.76	−0.90	10.19
SM_ColorM_b24 h	787	8.28	2.12	2.79	13.84	421	6.58	2.10	0.66	12.83
SM_ColorM_L24 h	787	42.70	3.36	33.72	53.20	421	44.25	3.39	33.83	52.92
SM_ColorScore_24 h (1–6)	794	3.60	0.84	1.00	5.50	421	3.36	0.61	1.50	5.00
**Drip loss** 4										
LM_DripEZ_24 h, %	794	1.11	0.48	0.22	5.25	423	2.50	1.91	0.13	8.74
LM_DripEZ_48 h, %	395	1.66	1.01	0.21	6.77	152	5.29	2.64	0.70	12.42
LM_DripBag_24 h, %	403	0.92	0.33	0.37	3.69					
SM_DripEZ_24 h, %	778	0.91	0.50	0.11	4.58	371	1.32	1.31	0.13	7.03
SM_DripEZ_48 h, %	396	1.09	0.54	0.22	3.22	127	3.23	1.99	0.55	9.17
**Chemical composition**										
LM_MoistureContent, %	876	74.45	1.51	61.50	86.94	421	74.75	0.91	70.12	78.94
LM_ProteinContent, %	511	22.20	1.29	11.95	34.82					
LM_IMF^5^, %	871	2.17	1.11	0.43	11.49	421	1.55	0.70	0.35	5.49
**Subjective scores**										
LM_Marbling (1–10)	794	1.98	0.77	1.00	10.00	421	2.23	0.55	1.00	4.00
SM_Marbling (1–10)	794	1.39	0.46	1.00	4.00	421	1.94	0.40	1.00	3.00
LM_Firmness (1–5)	406	3.00	0.59	1.00	4.50	277	2.81	0.53	1.50	4.50

1 Standard deviation.

2 pH measurements were taken on samples of the LM and SM at 45min and 24 h postmortem.

3 Color parameters a* (redness), b* (yellowness) and L* (lightness) were determined by a CM-2500/2600d Minolta Chroma Meter at 24 h postmortem. Meanwhile, subjective color score was recorded.

4 Drip loss of the LM and SM after 24 h or 48 h storage were measured using a bag method [24] and/or an EZ-DripLoss method [25].

5 Intramuscular fat content.

### Genotyping and Quality Control

Genomic DNA was isolated from ear clip or spleen tissues using a routine phenol/chloroform extraction method, and DNA concentration was diluted to 50 ng/ul. The quality and concentration of genomic DNA fulfilled the requirements for the Illumina Infinium SNP genotyping platform. Genotyping of 62,163 SNPs on the Illumina Porcine 60 K SNP Beadchip was carried out at the Illumina-certified service provider, Beijing Emei Tongde Technology Development Co. Ltd (EMTD). Genotypic data is available on all F2 and Sutai offsprings phenotyped, as well as their parents and/or grandparents. Quality control was carried out using PLINK v1.07 [28] for each population separately. SNP markers were removed if they had genotype-missing rates >0.03 or minor allele frequencies (MAF) <0.05 or Hardy-Weinberg *P*< = 10^−5^ (based on Chi-squared test). Samples were removed on low (<90%) call rate. After quality control, all samples passed the filter and a final set of 39,414 SNPs and 44,532 SNPs was selected for GWA in the F2 and Sutai populations, respectively. The distribution of SNP markers after filtering and marker density on each chromosome are shown in Table S1. Genotype data are deposited in the Dryad repository (http://dx.doi.org/10.5061/dryad.7 kn7r).

### Statistical Analyses

The association analyses were conducted using GenABEL in the R software [29]. SNPs were individually tested for association with all studied traits using a generalized linear mixed model. The model includes a random polygenic effect for which the variance-covariance matrix is proportional to genome-wide identity-by-state (IBS). The model equation is shown below:
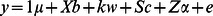
where *y* is the vector of phenotypes of all genotyped and phenotyped F2 or Sutai piglets; *μ* is the overall mean; *b* is the vector of fixed effects including sex and batch effects; *w* is the vector of slaughter weight of individuals considered as covariate; *c* is the vector of SNP effects with Erhualian allele substitute to White Duroc allele; *a* is the vector of random additive genetic effects with *α*∼N(0, Gσ_α_^2^), where G is the genomic relationship matrix calculated from the corrected pedigree and σ_α_^2^ is the polygenetic additive variance); *k* is the regression coefficient of slaughter weight and *e* is the vector of residual errors with *e*∼N(0, Iσ_e_^2^), where *I* is the identity matrix and σ_e_^2^ is the residual variance. X, S and Z are incidence matrices for *b*, *w* and *c* respectively. The herd-year-season effect was contained in the batch effect.

The genome-wide significance threshold was determined by the Bonferroni method, in which the conventional *P-*value was divided by the number of tests performed [30]. A SNP was considered to have genome-wide significance at *P*<0.05/N and have chromosome-wide significance at *P*<1/N, where N is the number of SNPs tested in the analyses. The genome-wide and chromosome-wide significant thresholds were 1.27e-6 (0.05/39414) and 2.54e-5 (1/39414) respectively for the F2 population, and were 1.12e-6 (0.05/44532) and 2.25e-5 (1/44532) respectively for the Sutai population.

**Figure 1:**
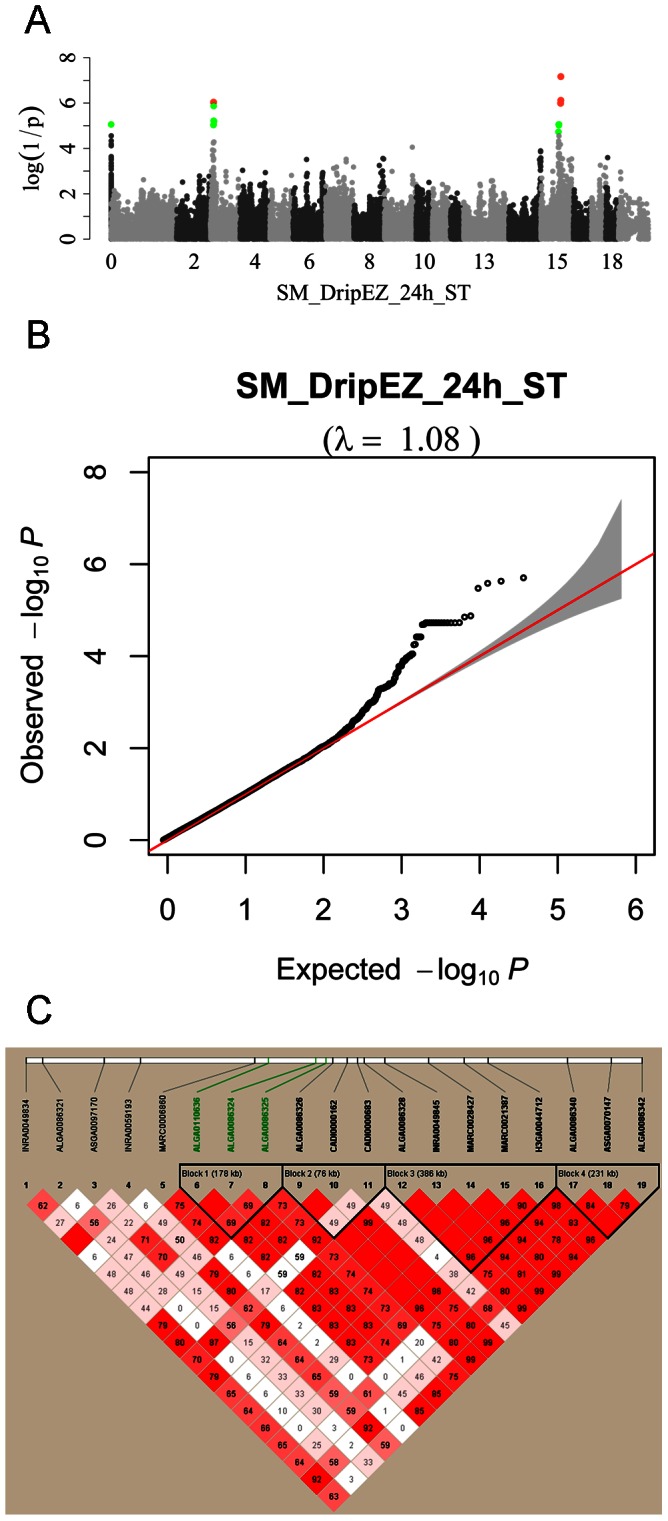
Genome-wide association results for the drip loss after 24 h storage of semimembranosus muscle (SM) from the Sutai (ST) population (SM_DripEZ_24 **h_ST).** (**A**) Manhattan plot showing the significance of association between 43760 SNPs and the drip loss trait. The red and blue dots represent the SNPs that reached a genome-wide significance level (*P*<1.12×10^−6^) and a chromosome-wide significance (*P*<2.25×10^−5^), respectively. There are three genome-wide significant SNPs (ALGA0086325, ALGA0086324 and ALGA0110636) on SSC15. (**B**) Quantile-quantile plot for this trait. The horizontal axis indicates the expected −log_10_(P-values) and the vertical axis indicates the observed −log_10_(P-values). The diagonal line represents y = x, which corresponds to the null hypothesis, and the shaded region shows 95% confidence interval based on Beta distribution [73]. (**C**) Haplotype blocks on a 2-Mb region on SSC15 containing all genome-wide significant SNPs (in green) associated with the drip loss trait.

The influence of population stratification was assessed by examining the distribution of test statistics generated from the thousands of association tests and assessing their deviation from the null distribution (that expected under the null hypothesis of no SNP associated with the trait) in a quantile-quantile (Q-Q) plot [6]. In these plots (Figure 1B and Figure S2), –log_10_ *P* values for each SNP calculated from their observed association statistics (χ^2^ statistics) were ranked in order from smallest to largest on the y-axis and plotted against the distribution that would be expected under null hypothesis of no association on x-axis. Deviations from the diagonal identity line suggest that either the assumed distribution is incorrect or that the sample contains values arising in some other manner, as by a true association [31]. The Q-Q plot was constructed using R software.

Haplotype or linkage disequilibrium (LD) block analyses were performed for the chromosomal regions with multiple significant SNPs clustered around the peak SNP. The LD blocks were determined using Haploview version 4.2 software with default settings [32].

## Results

### Population Stratification Assessment

Population stratification for GWAS can lead to false positive results [6]. The Q-Q plots of the test statistics in GWA are shown in Figures 1B and S2. From these plots, it is apparent that there is no clear overall systematic bias in all studied traits. The genomic inflation factors (λ) observed in the GWA study were usually less than 1.10, also indicating that no very strong stratification existed.

### GWAS Analyses

Both genome-wide significant SNPs and chromosome-wide significant SNPs for the pH, meat color, drip loss, chemical compositions, marbling and firmness are presented in Tables 2, 3, 4, 5. The profiles of the *P*-values of the tested SNPs for all meat quality traits are shown in Figure 1A and Figure S1. In total, 127 chromosome-wide significant SNPs were identified and among them, 11 showed genome-wise significant association (with underlined *P*-value in the tables) with different traits: 1 for pH, 4 for meat color, 4 for drip loss and 2 for IMF.

**Table 2:** Description of SNPs significantly associated with pH values.

Pop^1^	Traits^2^	PeakSNP	No.^3^	Chr^4^	Pos (bp)^5^	Nearestgenes^6^	Alleles	FA_D^7^	FA_E^8^	FA_F2^9^	FA_ST^10^	Effect^11^	*P*-value^12^
F2	LM_pH 45 min	DRGA0003797	1	3	14,409,638	*ENSSSCG00000007727*	A/C	1	0.68	0.76		0.106	1.28E-05
	LM_pH 24 h	MARC0055594	1	X	142,047,331	*BCAP31*	A/G	1	0.56	0.79		−0.045	1.93E-05
	LM_pHdrop_45 min_24 h	DRGA0003797	1	3	14,409,638	*ENSSSCG00000007727*	A/C	1	0.68	0.76		0.106	7.53E-06
	SM_pHdrop_45 min_24 h	MARC0088806	1	2	8,200,317	*CHRM1*	A/C	0.5	0.41	0.44	0.20	−0.093	1.54E-05
	SM_pHdrop_45 min_24 h	ASGA0055704	1	13	3,670,164	*OXNAD1*	A/G	0.25	0.41	0.32	0.55	0.101	1.08E-05
ST	SM_pH 45 min	ALGA0026555	2	4	96,114,909	*ENSSSCG00000022220*	A/G	0.75	0.03	0.41	0.46	0.168	2.24E-05
	SM_pH 24 h	ASGA0089100	1	0	0		A/G	0	0.21	0.13	0.07	0.159	1.43E-05
	SM_pH 24 h	ASGA0094824	7	3	16,518,098	*C7ORF42*	A/G	0	0.82	0.44	0.23	0.107	8.44E-07
	SM_pHdrop_45 min_24 h	ASGA0089930	2	3	16,473,829	*C7ORF42*	A/C	0.75	0.47	0.69	0.61	0.212	1.46E-06

1 The White Duroc**×**Erhualian F2 population and Sutai (ST) population.

2 Description of the traits is available in Table 1.

3 The number of significant SNPs within the QTL regions.

4,5 SNPs position on the *Sus Scrofa* Build 10.2 assembly.

6 Gene names starting with ENSSSCG represent Ensembl nomenclature while other gene symbols represent HUGO nomenclature.

7,8,9,10 The SNP allele “A” frequencies of two F0 Duroc (FA_D), 17 F0 Erhualian (FA_E), the whole F2 population (FA_F2) and Sutai population (FA_ST).

11 Additive effects; positive value indicates that allele “A” increased the trait.

12 Genome-wide significant associations are underlined.

**Table 3:** Description of SNPs significantly associated with meat color.

Pop^1^	Traits^2^	Peak SNP	No.^3^	Chr^4^	Pos (bp)^5^	Nearest genes^6^	Alleles	FA_D^7^	FA_E^8^	FA_F2^9^	FA_ST^10^	Effect^11^	*P*-value^12^
**F2**	LM_ColorM_a24 h	ALGA0024582	1	4	38,015,849	*NCALM*	A/G	0	0.68	0.36	0.16	0.383	5.29E-06
	LM_ColorM_a24 h	MARC0006685	1	6	22,591,224	*ENSSSCG00000028630*	A/G	0	0.18	0.08	0.26	−0.543	2.04E-05
	LM_ColorScore_24 h	ALGA0073833	1	13	210,866,603	*TTC3*	A/G	0	0.47	0.30	0.38	0.151	2.07E-05
	SM_ColorM_L24 h	ALGA0039930	4	7	31,270,305	*LRRC1*	A/G	1	0.12	0.53		−0.976	8.42E-06
**ST**	LM_ColorM_a24 h	ALGA0060775	1	0 (11^13^)	0		A/G	0	0.12	0.09	0.05	1.636	2.07E-08
	LM_ColorM_a24 h	ASGA0049740	1	0 (11)	0		A/G	1	0.88	0.91	0.94	−1.414	5.81E-08
	LM_ColorM_a24 h	M1GA0014909	1	0 (11)	0		A/C	0.5	0.88	0.65	0.94	−1.457	3.71E-08
	LM_ColorM_a24 h	ASGA0103866	1	0	0		A/G	1	0.5	0.62	0.94	−0.895	5.01E-06
	LM_ColorM_a24 h	ASGA0053450	1	12	15,720,339	*TANC2*	A/G	0.75	0	0.30	0.09	0.806	1.89E-05
	LM_ColorM_b24 h	ALGA0016105	1	2	138,402,066	*HINT1*	A/G	0.5	0.03	0.23	0.83	0.648	2.24E-05
	LM_ColorM_b24 h	H3GA0023987	1	7	134,562,880	*GCM1*	A/G	0.5	0.94	0.70	0.08	1.075	1.87E-06
	LM_ColorM_b24 h	H3GA0041110	1	14	86,363,610	*KCNMA1*	A/G	0	0.38	0.21	0.35	0.617	1.90E-05
	LM_ColorScore_24 h	ALGA0057003	2	10	12,020,850	*ENSSSCG00000010825*	A/G	0.75	0.85	0.84	0.81	0.275	4.89E-06
	SM_ColorM_a24 h	ALGA0060775	1	0	0		A/G	0	0.12	0.09	0.05	1.546	2.05E-05
	SM_ColorM_a24 h	ALGA0040423	2	7	34,103,417	*TMEM217*	A/G	0	0.24	0.08	0.11	1.220	7.74E-07
	SM_ColorM_a24 h	ALGA0105452	1	10	72,861,788	*KLF6*	A/G	0.75	0.26	0.48	0.93	−1.229	1.63E-06
	SM_ColorM_L24 h	DRGA0005419	1	5	4,824,007	*SLC25A17*	A/G	1	0.21	0.60	0.83	−1.377	2.08E-05
	SM_ColorScore_24 h	ALGA0032052	1	5	60,978,291	*ARHGDIB*	A/G	0.5	0	0.26	0.05	0.440	1.46E-05

See footnotes in Table 2.

13 The Illumina PorcineSNP60 BeadChip map shows that the SNP is located on chromosome 11.

**Table 4:** Description of SNPs significantly associated with drip loss.

Pop^1^	Traits^2^	Peak SNP	No.^3^	Chr^4^	Pos (bp)^5^	Nearest genes^6^	Alleles	FA_D^7^	FA_E^8^	FA_F2^9^	FA_ST^10^	Effect^11^	*P*-value^12^
**F2**	LM_DripBag_24 h	H3GA0000077	10	1	1,614,750	*THBS2*	A/C	0.75	1	0.92	0.63	−0.216	4.50E-06
	SM_DripEZ_24 h	ASGA0020291	2	4	81,567,806	*FAM110B*	A/C	0.5	0.26	0.33		−0.120	2.04E-05
**ST**	LM_DripEZ_48 h	DRGA0005419	1	5	4,824,007	*SLC25A17*	A/G	1	0.21	0.60	0.83	−2.331	1.84E-05
	SM_DripEZ_24 h	ASGA0096756	1	0	0		A/G	0.25	0.12	0.22	0.10	0.937	8.69E-06
	SM_DripEZ_24 h	MARC0027412	1	0	0		A/G	0.25	0.18	0.29	0.10	0.937	8.69E-06
	SM_DripEZ_24h	H3GA0008920	5	3	15,772,472	*ENSSSCG00000007729*	A/G	0.25	0	0.12	0.64	0.538	9.16E-07
	SM_DripEZ_24 h	ALGA0086325	18	15	96,497,499	*ITGA4*	A/C	0.75	0.09	0.35	0.06	1.116	6.74E-08
	SM_DripEZ_48 h	ALGA0043720	1	0	0		A/G	0	0.09		0.18	1.721	1.54E-05
	SM_DripEZ_48 h	ASGA0090490	1	0	0		A/C	0	1	0.50	0.76	−1.908	1.07E-05
	SM_DripEZ_48 h	H3GA0015667	2	5	10,925,920	*SNORA50*	A/G	0.5	0	0.26	0.15	2.134	1.32E-05

See footnotes in Table 2.

**Table 5:** Description of SNPs significantly associated with moisture content, protein content and intramuscular fat content (IMF), and subjective scores of marbling and firmness.

Pop^1^	Traits^2^	Peak SNP	No.^3^	Chr^4^	Pos (bp)^5^	Nearest genes^6^	Nearby Gene^13^	Alleles	FA_D^7^	FA_E^8^	FA_F2^9^	FA_ST^10^	Effect^11^	*P*-value^12^
**F2**	LM_MoistureContent	MARC0033464	6	7	35,177,641	*SPDEF*	*HMGA1* (B)	A/G	0	0.88	0.45	0.06	0.475	9.49E-06
**ST**	LM_MoistureContent	MARC0009151	1	10	8,270,861	*ESRRG*		A/G	0.5	0.12	0.28	0.05	−0.498	8.26E-06
**F2**	LM_ProteinContent	MARC0058766	2	7	34,803,564	*GRM4*		A/G	0	0.94	0.46	0.06	−0.430	2.09E-05
**F2**	LM_IMF	ALGA0043983	1	7	104,352,654	*FOS* (B, E)		A/G	0	0.41	0.23	0.08	0.296	2.48E-05
	LM_IMF	ALGA0067119	1	12	58,078,076	*TMEM220*	*MYH1* (B)*MYH2* (B),*MYH3* (B)	A/G	1	0.82	0.94	0.54	−0.441	2.30E-05
	LM_IMF	MARC0090296	2	X	46,124,768	*SLC9A7*	*RGN* (B)	A/C	1	0.76	0.90		−0.332	8.92E-07
	LM_IMF	ALGA0099852	3	X	103,627,248	*ENSSSCG00000012572*		A/G	0.5	0	0.20		−0.232	1.13E-05
**ST**	LM_IMF	ASGA0087693	1	0	0			A/G	1	1		0.90	−0.352	1.24E-05
	LM_IMF	ASGA0005433	2	1	205,120,122	*DLGAP5*	ATG14 (B)	A/G	0.5	0.18	0.33	0.85	−0.322	4.74E-06
	LM_IMF	ALGA0049586	1	8	134,540,073	*PDLIM5*	*BMPR1B* (B)	A/G	0	0.44	0.21	0.10	0.346	2.17E-05
	LM_IMF	ALGA0053636	20	9	74,772,957	*ADAM22*	*STEAP4* (B)	A/G	1	0	0.49	0.31	0.251	1.12E-06
	LM_IMF	MARC0013398	1	9	152,109,779	*ENSSSCG00000015634*		A/G	0	0.32	0.20	0.14	0.273	2.25E-05
**F2**	SM_Marbling	MARC0090739	1	13	216,093,269	*TFF1*	*UBASH3A* (M)	A/G	0.25	0.74	0.54	0.35	0.109	1.51E-05
**F2**	LM_Firmness	MARC0058766	2	7	34,803,564	*GRM4*	*LEM2* (B),*HMGA1* (B)	A/G	0	0.94	0.46	0.06	−0.255	6.42E-06

See footnotes in Table 2.

13 Candidate genes within 500 kb upstream and downstream of the peak SNP. B, biological candidate; E, gene expressed differentially in muscle among pig breeds; M, gene DNA methylation level changes.

#### pH values

Five and seven SNPs significantly associated with pH traits were identified in the F2 and Sutai pigs respectively (Table 2). All the SNPs except for unmapped markers represent five QTL regions on SSC2, 3, 4, 13 and X. The QTL region on SSC3 was common to the two populations. This region from 14.4 Mb to 17.4 Mb harbors one SNP (DRGA0003797) associated with both pH 45 min and pH drop from postmortem 45 min to 24 h of LM in the F2 population, and 9 SNPs associated with both pH 24 h and pH drop of SM in the Sutai population. No significant SNPs were found for pH values of LM in the Sutai population.

#### Meat color

We identified 8 and 16 significant SNPs associated with meat color in the F2 population and the Sutai population respectively (Table 3). No common QTL region for the same trait was detected in the two populations. However, SNP ALGA0039930 at 31.27 Mb on SSC7 that was associated with Minolta L* of SM in the F2 population is adjacent to another SNP ALGA0040423 at 37.73 Mb that showed significant association with Minolta a* of SM in the Sutai population. The most significant SNP associated with Minolta a* of both LM and SM in Sutai was the SNP ALGA0060775. This SNP reached genome-wide significance level and was located very close to the other two genome-wide significant SNPs ASGA0049740 and MIGA0014909 for the same trait on chromosome 11.

#### Drip loss

In the F2 population, a total of 12 SNPs were detected to be significantly associated with drip loss of LM and SM after 24 h storage (Table 4). Ten out of these SNPs fall in the region of 1.53 Mb (from 1.31 Mb to 2.84 Mb) on SSC1, and the other two were located at 81.56 Mb and 81.63 Mb on SSC4. As for the Sutai population, there were 30 significant SNPs with effect on drip loss, out of which 3 on SSC15 and 1 on SSC3 reached genome-wide significance level for drip loss of SM after 24 h storage (Fig. 1A). The three most significant SNPs ALGA0086325 (*P* = 6.74E-08), ALGA0086324 (*P* = 7.64E-07) and ALGA0110636 (*P* = 1.03E-06) on SSC15 were in a haplotype block spanning 178 kb (Fig. 1C).

#### Moisture, protein and IMF contents, marbling and firmness scores

Forty-four SNPs were significantly associated with these traits: 7 for moisture content of LM, 2 for protein content of LM, 32 for IMF of LM, 1 for marbling of SM and 2 for firmness of LM (Table 5). In the F2 population, a 0.46-Mb region from 34.80 Mb and 35.26 Mb on SSC7 contains not only 6 SNPs associated with moisture content of LM, but also 2 SNPs associated with protein content and 2 SNPs associated with firmness of LM. Of the 32 SNPs associated with IMF of LM, 7 were detected in the F2 animals with the most significant SNP MARC0090296 on SSCX (*P* = 8.92E-07), and 25 in the Sutai pigs with the top SNP ALGA0053636 on SSC9 (*P* = 1.12E-06). Neither common loci for IMF nor significant SNPs associated with marbling of LM were found in the two populations. Only one SNP MARC0090739 on SSC13 showed a significant association with marbling of SM in the F2 population.

## Discussion

To our knowledge, only one study [16] has applied GWA approach to detect QTL signals for IMF, marbling, meat color and moisture in a Large White**×**Minzhu F2 population. This article reported that most significant SNPs (except for unmapped SNPs) for these traits were located within a 10.70 Mb region (51.37–61.07 Mb) on SSC12. In this region, we also identified a chromosome-wide significant SNP ALGA0067119 at 58.08 Mb for IMF of LM. The favorable allele (G) that increases IMF derived from Erhualian (Table 5). Whereas our results did not confirm the associations between this region and other phenotypes, and demonstrated that generally more than one genomic region are associated with meat quality traits.

### Previous and Novel QTLs

Using the GWA analyses of 933 F2 individuals and 434 Sutai piglets, we herein identified 127 SNPs significantly associated with the 25 pork quality traits. Of these SNPs, 116 were located in 28 genomic regions of approximately 10 Mb or less, while others cannot be mapped to the current pig genome assembly (*Sus Scrofa* Build 10.2). Most of the SNP effects overlapped with previously reported QTL regions on SSC2, 3, 4 and 13 for pH [18], [33]–[37], on chromosomes 2, 4, 5, 6, 7, 10, 13 and 14 for color [19], [37]–[45], on SSC1, 3, 4, 5 and 15 for drip loss [46]–[49], on SSC7 for moisture content [19], [50] and protein contents [40], on SSC1, 7, 9, 12 and X for IMF [19], [51], [52], on SSC13 for marbling [19]. Furthermore, this GWA study revealed 7 novel loci: Three were found in the F2 population, including SNPs MARC0058766 for firmness score of LM on SSC7, MARC0055594 for pH 24 h of LM on SSCX and ALGA0099852 for IMF of LM on the same chromosome; The remaining 4 SNPs were identified in the Sutai population, including H3GA0023987 on SSC7 for color b* of LM, ALGA0049586 on SSC8 for IMF of LM, and MARC0009151 on SSC10 for LM moisture content and ALGA0057003 on the same chromosome for LM color score.

### Possible Pleiotropic QTLs

The present results showed that several regions contain multiple significant SNPs associated with different traits. Especially, the SSC7 region from 31.27 Mb to 37.74 Mb harbored SNPs affecting five traits: MARC0069646 for color parameter a*, ALGA0039930 for color parameter L*, MARC0033464 for moisture content, MARC0058766 for protein content and firmness. Our previous QTL mapping study [19] also demonstrated that this region have strong QTL effects on various carcass and meat quality traits measured in the F2 population. So the current GWA result is consistent with the result of linkage analysis. Moreover, the GWA study enhanced the precision of QTL mapping. For example, all 6 significant SNPs associated with moisture content fell into a 0.46 Mb region (34.80–35.26 Mb) on SSC7, much smaller than previously reported QTL interval of 12 cM (approximately 12 Mb).

Additionally, a 3.01 Mb region on SSC3 (from 14.40 Mb to 17.41 Mb) was found to be associated with both pH values (pH 45 min, pH 24 h and pH drop from postmortem 45 min to 24 h) and drip loss. Because the development of drip loss is largely governed by the rate and extent of postmortem pH decline [53], it is likely that there is a common causative variant for these related traits within the region. Similarly, a common SNP DRGA005419 on SSC5 is associated with both L* value of SM and DripEZ_48 h of LM in the Sutai piglets. Combined with the correlation coefficient of 0.45 (significantly greater than zero, *P*<0.01) between the two traits, it suggests the existence of a pleiotropic QTL simultaneously regulating meat color and drip loss.

### GWA QTLs vs. Linkage Mapping QTLs

Previously, a genome-wide significant QTL for IMF was mapped to a region flanked by microsatellite makers *SW2456* and *S1426* (48–58 cM and 42–103 Mb) on SSCX in the F2 population [19]. This region has a very low recombination rate (average 6 Mb per cM) [54], making it very difficult to fine-map the QTL and to discriminate between multiple QTLs and single QTL by family-based linkage analysis. Fortunately, it is not a big challenge in GWA studies because it can capitalize on all meiotic recombination events in a population, rather than only those occurred currently in the studied families. It is, therefore, not surprising that the association signals for IMF were localized to two distant and small regions on SSCX in this study: one harboring 2 significant SNPs at 45.39 Mb and 46.12 Mb respectively, another harboring 3 significant SNPs from 103.62 Mb to 104.43 Mb. Moreover, the two regions also tended to be associated with marbling score (SNP MARC0090296 at 46.12 Mb with *P*-value of 2.74E-05 and SNP H3GA0051863 at 104.42 Mb with *P*-value 1.65E-04, approaching significance level), because IMF and marbling score are highly correlated (r = 0.71, *P*<0.01).

There are some differences in QTL findings between the present GWA study and the previously published genetic linkage studies using the same F2 population. Several 1% genome-wide significant QTLs that were reported in our previous papers [18], [19] failed to replicate in this study, such as two QTLs for IMF of LM on SSC9 and one for color score of SM on SSC11. Such discrepancy maybe due to the following reasons: (1) Linkage analyses were performed under an assumption that the founder lines are fixed for different QTL alleles, whereas GWA analyses systematically investigate SNPs in the entire genome without the constrains of a priori hypotheses; (2) Additive, dominant and even imprinting effects of putative QTL were estimated in the linkage study, whereas only additive effect was tested in this GWA study; (3) We used the QTL linkage mapping procedure suggested by Guo et al. (2008) [55]. This procedure obtained estimates by fitting all identified QTLs as genetic background effects at each step of searching new QTL. In contrast, the linear mixed model was fixed in the GWA study; (4) We used a permutation method and a Bonferroni method to determine the significance thresholds for linkage mapping analysis and GWA analysis respectively. Compared with the permutation method, the Bonferroni correction method operates too conservative, because it assumes the independence of each test even though many of the SNPs are in linkage disequilibrium (LD) and thus correlated with each other. As a result, the Bonferroni power to detect some statistically significant results became relatively weak.

### Common and Population-specific QTLs

Repeated detection of a QTL among populations is a way to validate the QTL. Interestingly, the GWA analyses of the Sutai population revealed some genome-wide significant SNPs for IMF and color a* on SSC9 and 11 respectively. They situate within the above-mentioned QTL regions detected in the F2 population. This result thus provided evidence that those genome-wide significant QTLs found in the F2 population are unlikely to be artifacts of linkage analyses.

In addition, several association signals, e.g. those for pH values on SSC3 and for color parameters on SSC7 were repeatly identified in the two populations, validating the existence of these loci. However, many association signals appeared in only one population. This maybe resulted from the differences in environmental background (such as birthplaces, times of weaning and castration, and etc.) and genetic background (because of founder lines, population structure, selection, gene-gene interactions, and etc.) between the two populations.

### Candidate Genes

We noticed that the significant SNPs for pH, meat color, drip loss, moisture content and protein content are rarely situated within or near known genes affecting these traits. Only one SNP DRGA0005419 for drip loss on SSC5 was located 436 kb upstream of the *ADSL* (*adenylosuccinate lyase*) gene that was found to be possibly associated with drip loss and pH 45 min of LM in Pietrain pigs [56].

In contrast, according to gene biological functions in lipid metabolism, adipocyte and/or muscle development, we identified some candidate genes for IMF, marbling and firmness within 500 kb upstream/downstream of the peak SNPs. In the F2 population, four GWA QTLs for IMF were detected on SSC7, SSC12 and SSCX. The *FOS* gene is closest to the significant SNP ALGA0043983 on SSC7. This gene encodes a leucine zipper protein that has been implicated as a regulator of signal transduction, cell proliferation and differentiation (e.g. myogenesis) [57]. Furthermore, it was found to be expressed differentially in muscle between the fat type pig breeds (such as Basque and Liangtang) and lean type pig breeds (Large White and Landrace) [58]. Therefore, the *FOS* gene could be regarded as a prime candidate gene for the QTL. Within the QTL region on SSC12, the *MYH1*, *MYH2* and *MYH3* genes that belong to the myosin heavy chain gene family (MYH) have been proposed as candidate genes by Luo et al. (2012), who also identified this QTL in their GWA study. On SSCX, a genome-wide significant SNP MARC0090296 for IMF is located at 46.12 Mb, within the *SLC9A7* (S*olute carrier family 9 member 7*) gene. A promising gene, *RGN* (also called as *SMP30*, i.e. *regucalcin* or *senescence marker protein-30*), is located 438 kb away from this SNP. Regucalcin plays a multifunctional role as a regulatory protein in intracellular signaling processes in many cell types and is related to lipid metabolism [59]. Regucalcin transgenic rats have been shown to experience hyperlipidemia with increasing age [60]. No apparent candidate genes are located in the vicinity of the SNP ALGA0099852 at 103.62 Mb on SSCX.

In the Sutai population, we found four QTLs for IMF on SSC1, SSC8 and SSC9. A candidate gene for the QTL on SSC1 is the *ATG14* (*autophagy related 14 homolog*) gene that plays an important role in hepatic lipid metabolism [61]. The QTL effect detected on SSC8 could be due to the candidate gene *BMPR1B* [*bone morphogenetic protein* (BMP) *receptor, type IB*], because the ligands of this repceptor is BMPs that can induce commitment of C3H10T1/2 pluripotent stem cells into adipocytes [62], [63]. The *STEAP4* gene encoding metalloreductase, which is associated with obesity and insulin-resistance in human [64]–[66], is located at 74.98 Mb on SSC9, very close to the strongest association signal (ALGA0053636) detected in the Sutai piglets, and thereby is an excellent positional and biological candidate gene for this QTL. No obvious candidate genes for IMF were found in the distal region (around 152.11 Mb) of SSC9.

The peak SNP MARC0090739 for marbling score is located only 55 kb from the *UBASH3A* gene, which has a role in immune function and was observed to be differentially methylated in peripheral blood leukocytes between lean and obese adolescents [67]. On SSC7, the peak SNP MARC0058766 (at 34.80 Mb) for the firmness and moisture content, is also significantly associated with the protein content and drip loss of LM in the F2 population. The SNP is located between two candidate genes: the *LEM2* gene (at 34.64 Mb) and the *HMGA1* gene (at 34.98 Mb). The *LEM2* (also called *NET25*) gene is involved in nuclear structure organization and its mutations cause muscular dystrophies and other disorders [68]. The *HMGA1* gene encodes high mobility group AT-hook 1 protein that may play critical role in adipogenesis [69] and serve as a modulator of IGF-I activity [70]. The significant associations between polymorphisms in this gene and backfat thickness as well as drip loss have been reported [71], [72].

### Conclusions

In summary, this GWA study identified 11 genome-wise significant SNPs and 116 chromosome-wide significant SNPs associated with 25 meat quality traits. Our results narrow down the previously detected QTL intervals, and reveal 7 new QTL positions. At least two QTL regions on SSC3 and SSC7 were found to affect multiple traits and are common to the two populations. However, many QTLs are not conserved across the two populations, reflecting the genetic heterogeneity of these QTLs and the complexity of the genetic basis of pork quality. For some traits including pH values, drip loss and firmness, it is the first time that they are included in a GWA analysis. In the QTL regions, some candidate genes stand out because of their functional annotations, positions and reported expression variation in related tissues. The current findings will contribute to further identification of the causal mutation underlying these QTLs and future improvement of meat quality in pig breeding programmes.

## Supporting Information

Figure S1 **Manhattan plot of genome-wide association analysis for meat quality traits of longissimus muscle (LM) and semimembranosus muscle (SM) from a White Duroc×Erhualian F2 population and a Sutai (ST) population.** (**A**) pH phenotypes; (**B**) meat color phenotypes; (**C**) drip loss phenotypes; (**D**) chemical compositions (moisture, IMF, protein), marbling and firmness scores.
(RAR)

Figure S2 **Qiantile-quantile plot of SNPs after quality control in genome-wide association analysis for each meat quality trait.**
(TIFF)

Table S1 **Distribution of SNPs after quality control and average distances on each chromosome.**
(DOC)
