# Supplementary figures and images for: Genome-Wide Association Study of Meat Quality Traits in a White Duroc×Erhualian F2 Intercross and Chinese Sutai Pigs
Source: PLoS One. 2013 May 28;8(5):e64047. doi: 10.1371/journal.pone.0064047 (PMC3665833; doi:10.1371/journal.pone.0064047)

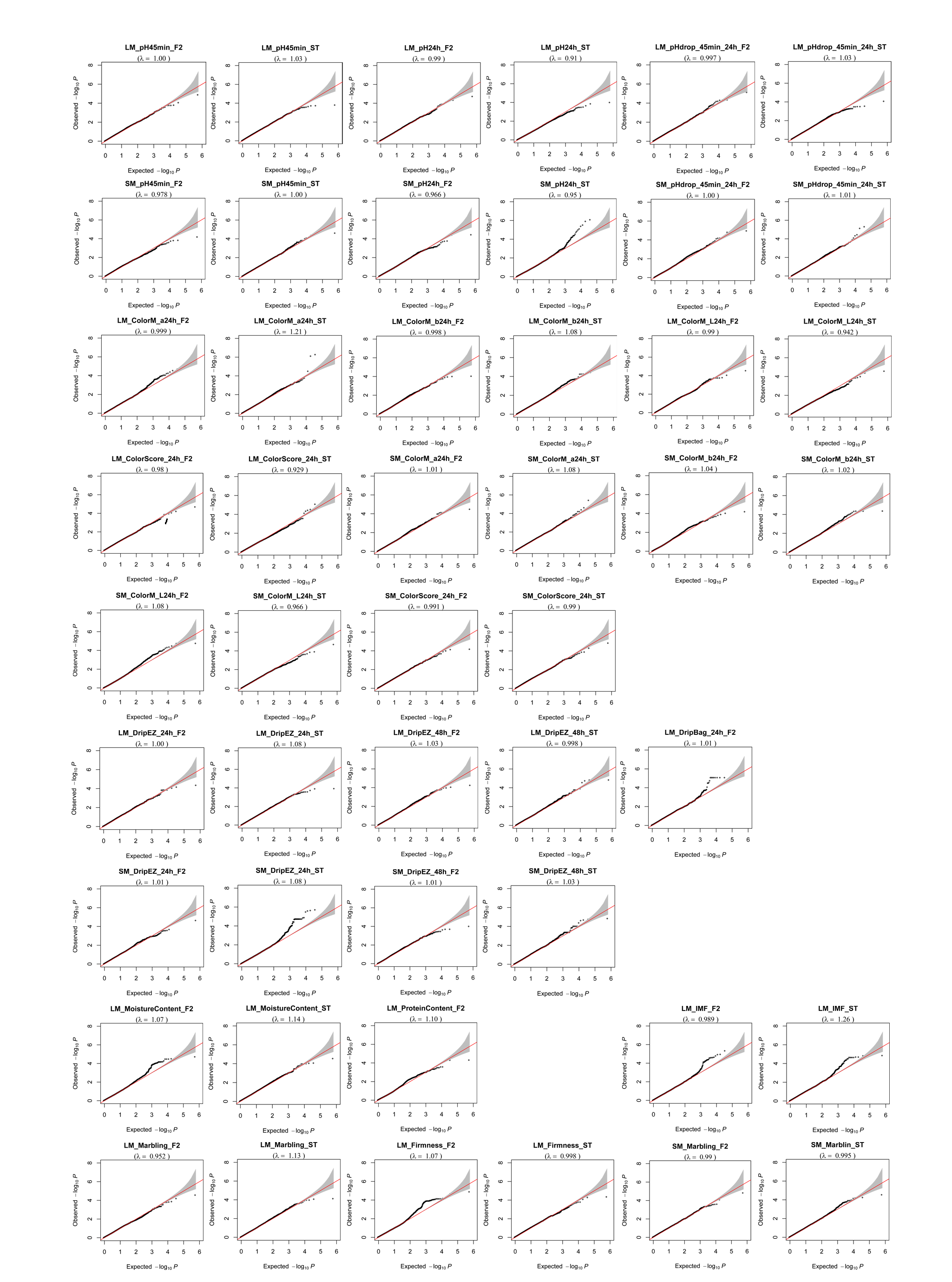

Supplement: Figure S2 — Qiantile-quantile plot of SNPs after quality control in genome-wide association analysis for each meat quality trait. (TIFF) [file pone.0064047.s002.tiff]
