# Supplementary material for: Genome-Wide Association Study of Meat Quality Traits in a White Duroc×Erhualian F2 Intercross and Chinese Sutai Pigs
Source: PLoS One. 2013 May 28;8(5):e64047. doi: 10.1371/journal.pone.0064047 (PMC3665833; doi:10.1371/journal.pone.0064047)
Supplement: Table S1 — Distribution of SNPs after quality control and average distances on each chromosome. (DOC) [file pone.0064047.s003.doc]

**Table S1.** Distribution of SNPs after quality control and average distances on each chromosome.

| **Chromosome** | **F2 population** | |  | **Sutai population** | |
| --- | --- | --- | --- | --- | --- |
|  | **No. of SNPs** | **Average distance (Kb)** |  | **No. of SNPs** | **Average distance (Kb)^a^** |
| 1 | 4184 | 75.26 |  | 4791 | 65.73 |
| 2 | 2274 | 71.36 |  | 2583 | 62.83 |
| 3 | 1883 | 76.39 |  | 2144 | 67.22 |
| 4 | 2396 | 59.84 |  | 2646 | 54.18 |
| 5 | 1419 | 78.24 |  | 1770 | 62.84 |
| 6 | 2066 | 76.10 |  | 2385 | 65.96 |
| 7 | 2237 | 60.19 |  | 2361 | 57.02 |
| 8 | 1811 | 81.53 |  | 2087 | 70.57 |
| 9 | 2052 | 74.83 |  | 2397 | 64.07 |
| 10 | 1112 | 69.98 |  | 1376 | 56.56 |
| 11 | 1369 | 63.99 |  | 1458 | 60.11 |
| 12 | 1051 | 60.16 |  | 1128 | 56.22 |
| 13 | 2677 | 81.48 |  | 2889 | 75.5 |
| 14 | 2708 | 56.73 |  | 2974 | 51.65 |
| 15 | 2007 | 78.43 |  | 2203 | 71.45 |
| 16 | 1306 | 66.33 |  | 1335 | 64.89 |
| 17 | 1192 | 58.13 |  | 1289 | 53.77 |
| 18 | 847 | 71.77 |  | 997 | 60.97 |
| X | 472 | 304.40 |  | 866 | 166.32 |
| Y | 1 |  |  | 2 |  |
| 0^b^ | 4350 |  |  | 4851 |  |
| Total | 39414 |  |  | 44532 |  |

^a^Derived from Sus scrofa genome assembly 10.2 (http://www.ensembl.org/Sus_scrofa/Info/Index).

^b^These SNPs are not assigned to the Sscrofa 10.2 genome assembly.
